# Supplementary material for: UPF1 promotes the formation of R loops to stimulate DNA double-strand break repair
Source: Nat Commun. 2021 Jun 22;12:3849. doi: 10.1038/s41467-021-24201-w (PMC8219777; doi:10.1038/s41467-021-24201-w)
Supplement: Supplementary file 1 — Supplementary Information [file 41467_2021_24201_MOESM1_ESM.pdf]

UPF1 promotes the formation of R loops to stimulate DNA double-strand break repair

Greg H.P. Ngo, Julia W. Grimstead and Duncan M. Baird

Supplementary information

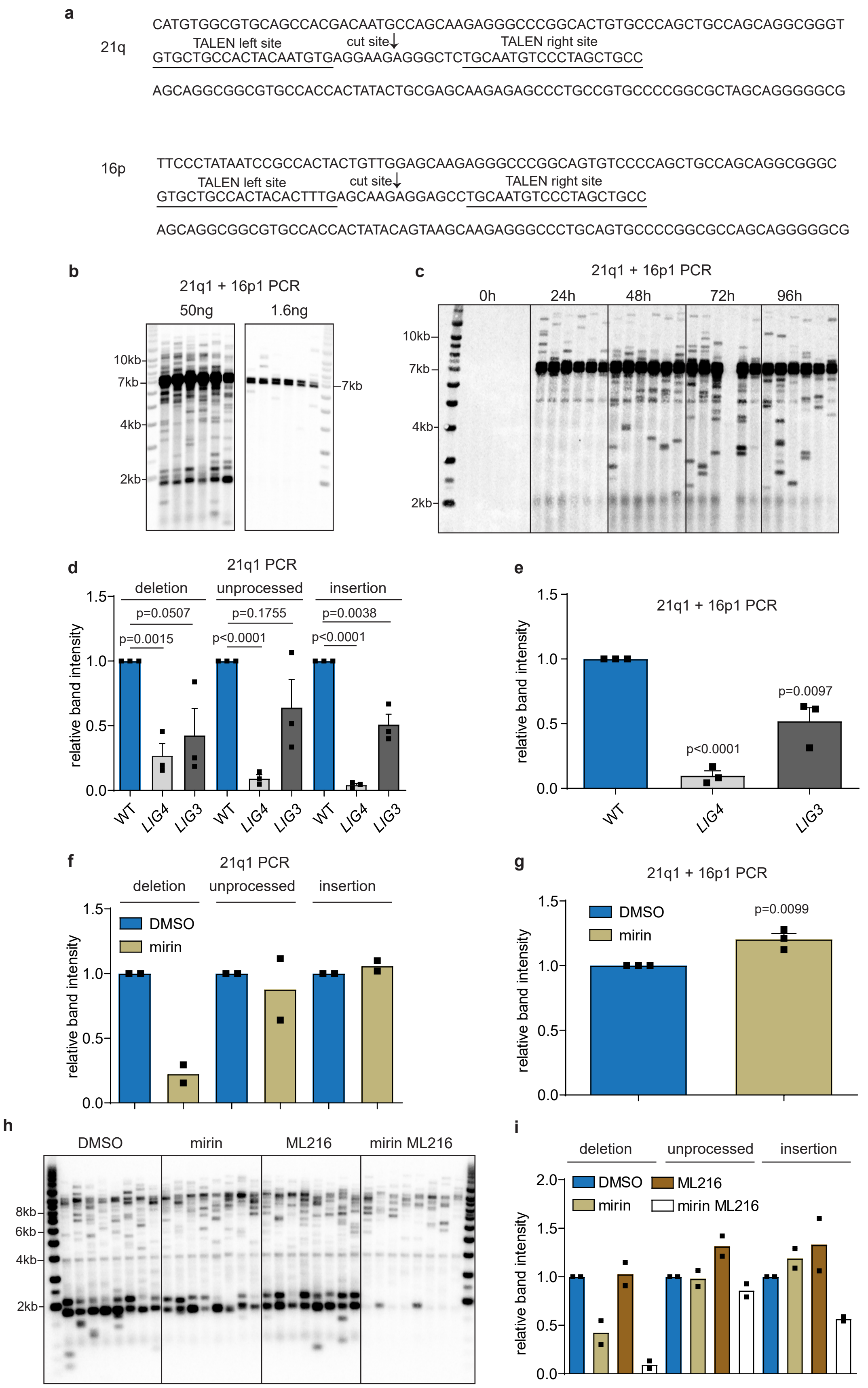

Supplementary Figure 1. DNA resection contributes to deletion formation at telomere fusion. a The DNA sequences at the 21q and 16p subtelomeres region (from centromere towards telomeres) are shown with the TALEN recognition and cutting sites indicated. **b** 21q1+16p1 telomere fusion analysis using the indicated amount of DNA isolated from RPE1-hTERT cells 48 hours after 21q/16p TALEN nucleofection. Telomere fusion products were detected with a 21q telomere adjacent probe. **c** 21q1+16p1 telomere fusion analysis using DNA (50ng) isolated from RPE1-hTERT cells at the indicated time after 21q/16p TALEN nucleofection. Telomere fusion products were detected with a 16p telomere adjacent probe. **d,e** Bar chart showing quantification of the intensity of fusion bands (21q1 PCR in d and 21q1+16p1 PCR in e) in HCT116 WT, *LIG4* KO, *LIG3* KO cells (relative to values in WT). Data plotted are mean+SEM (n=3). P values were obtained using Student's t-test (unpaired 2 tailed, equal variance). **f,g** Bar chart showing quantification of the intensity of fusion bands (21q1 PCR in f and 21q1+16p1 PCR in g) in RPE1-hTERT cells treated with DMSO or mirin (relative to values in DMSO). Data plotted are mean+SEM (n=2 for f, n=3 for g). P values were obtained using Student's t-test (unpaired 2 tailed, equal variance). **h** 21q1 telomere fusion assay using DNA isolated from RPE1 cells treated with DMSO, 50uM mirin or/and 50uM ML216 48 hours after nucleofection. **i** Bar chart showing quantification of the intensity of fusion bands in RPE1-hTERT cells treated with DMSO or the indicated inhibitors (relative to values in DMSO). Data plotted are means (n=2). P values were obtained using Student's t-test (unpaired 2 tailed, equal variance). The n values indicate the number of independent experiments in all cases.

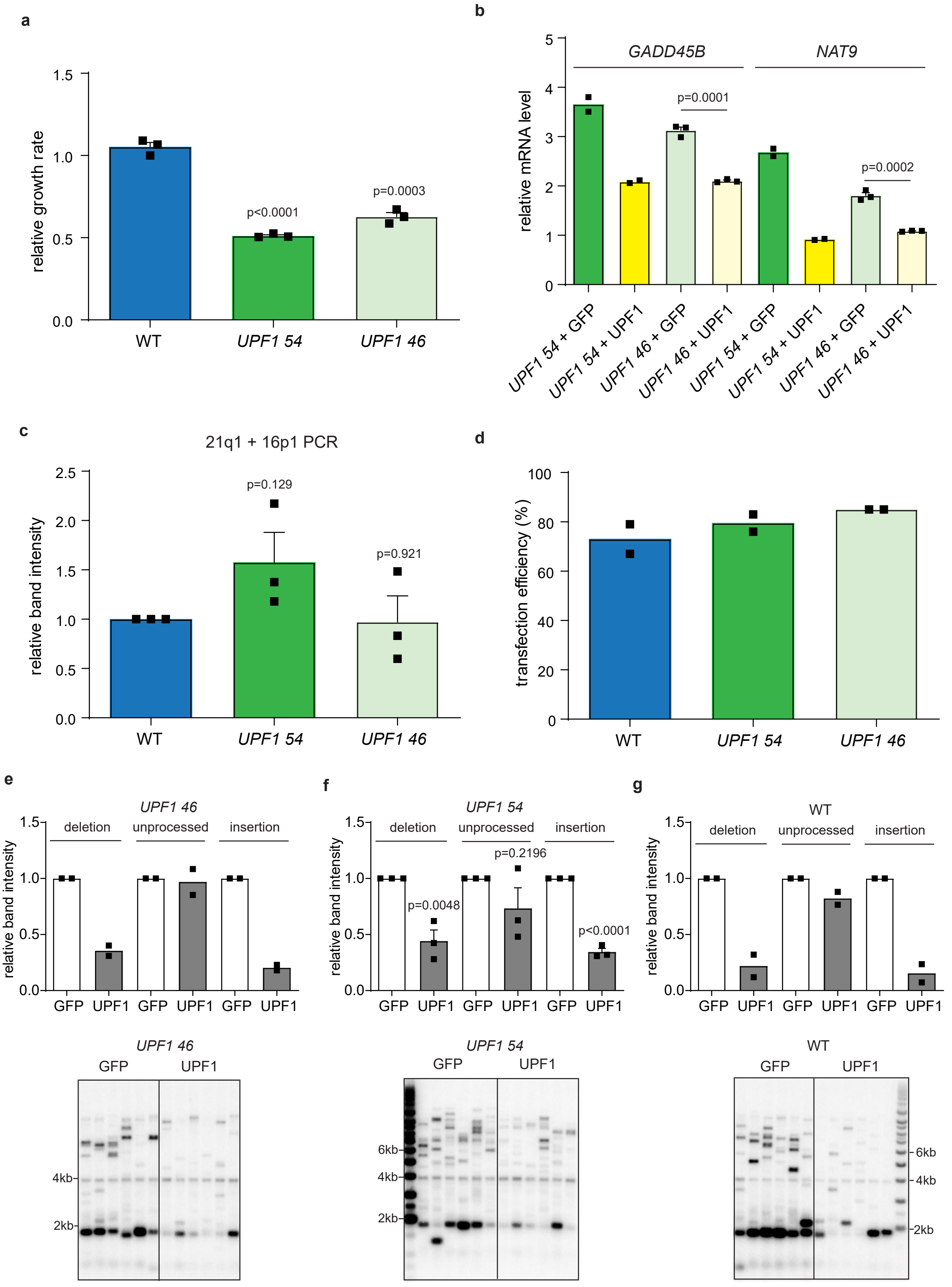

Supplementary Figure 2. UPF1 is involved in promoting telomere deletion. a Growth rate of RPE1-hTERT WT and *UPF1* KO cells (relative to the WT). Data plotted are means + SEM (n=3). P values were obtained using Student's t-test (unpaired 2 tailed, equal variance). **b** RT-qPCR analysis showing the mRNA level of two NMD markers *GADD45B* and *NAT9* in *UPF1* KO cells expressing UPF1 or GFP control. The values shown are relative to the values in WT cells and normalised with the mRNA level of *GAPDH*. Data plotted are means + SEM (n=3 for UPF1 46, n=2 for UPF1 54). P values were obtained using Student's t-test (unpaired, 2 tailed, equal variance). **c** Bar chart showing quantification of the intensity of fusion bands in RPE1-hTERT WT and *UPF1* KO cells (relative to values in WT). Data plotted are mean+SEM (n=3). P values were obtained using Student's t-test (unpaired 2 tailed, equal variance). **d** Transfection efficiency of RPE1-hTERT WT and *UPF1* KO cells. Data plotted are means (n=2). **e-g** 21q1 telomere fusion assay using DNA isolated from RPE1 WT or *UPF1* KO cells expressing UPF1 or GFP control and bar chart showing quantification of the intensity of fusion bands. Data plotted are mean+SEM (n=2 for e and g, n=3 for f). P values were obtained using Student's t-test (unpaired 2 tailed, equal variance). The n values indicate the number of independent experiments in all cases.

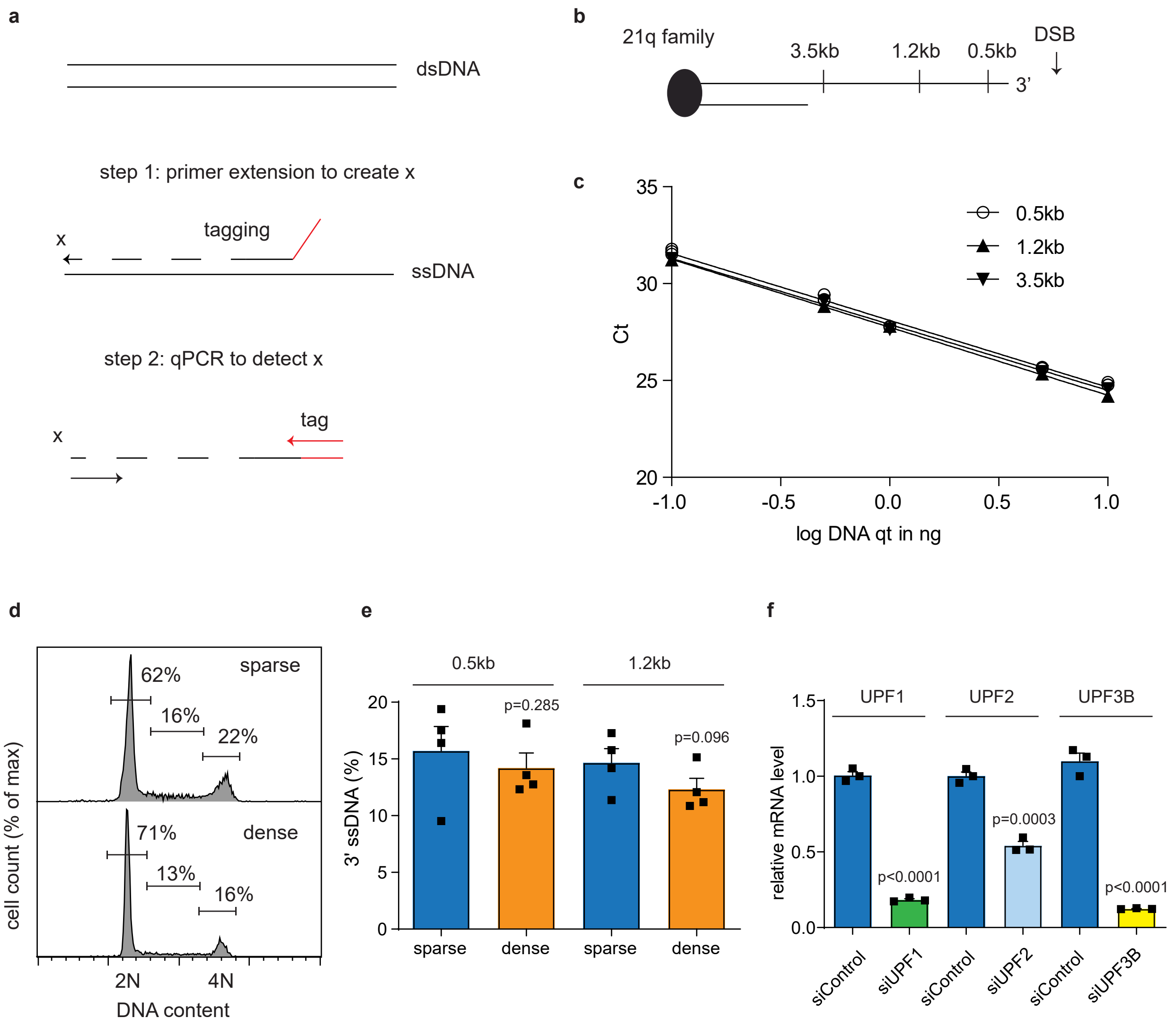

Supplementary Figure 3. UPF1 stimulates DNA resection at sub-telomeric DSBs independently of NMD. **a** Diagram showing how QAOS assay quantifies ssDNA. In step one, a tagging primer, containing locus specific sequence and an artificial tag sequence, binds to ssDNA but not to dsDNA. Ex Taq polymerase extends the tagging primer to create a hybrid with the tag sequence incorporated during a slow ramp from 40 to 72°C. The amount of this tagged hybrid is proportional to the initial amount of ssDNA. In step two, this hybrid sequence is quantified using stringent qPCR reaction containing the tag primer, a forward/reverse primer and a Taqman probe. **b** Diagram showing QAOS assay to detect 3′ ssDNA at three loci located at 0.5kb, 1.2kb and 3.5kb from TALEN cleavage site. **c** Threshold cycle (Ct) values were obtained from QAOS qPCR for the indicated loci and plotted against the log value of the amount of boiled DNA to generate a standard curve. **d** Cell cycle analysis of RPE1-hTERT WT cells (sparse or dense) with the level of G1, S and G2/M cells indicated. **e** QAOS analysis showing the level of 3′ssDNA at the indicated loci in RPE1-hTERT WT cells (sparse or dense) at 10 hours after 21q/16p TALEN nucleofection. Data plotted are means + SEM (n=4). P values were obtained using Student's t-test (unpaired 1 tailed, equal variance). **f** RT-qPCR analysis showing the mRNA level of *UPF1/UPF2/UPF3B* in RPE1-hTERT WT cells transfected with UPF1, UPF2, UPF3B or control siRNAs. The values shown are relative to the values in siControl and normalised with the mRNA level of *GAPDH*. Data plotted are means + SEM (n=3). P values were obtained using Student's t-test (unpaired, 2 tailed, equal variance). **g** QAOS analysis showing the level of 3′ssDNA at the indicated loci in RPE1-hTERT WT cells treated with DMSO or NMDI14 at 10 hours after 21q/16p TALEN nucleofection. Data plotted are means + SEM (n=3). P values were obtained using Student's t-test (unpaired 1 tailed, equal variance). **h** RT-qPCR analysis showing the mRNA level of two NMD markers *GADD45B* and *NAT9* in RPE1-hTERT WT cells treated with DMSO or NMDI14. The values shown are relative to the values in DMSO and normalised with the mRNA level of *GAPDH*. Data plotted are means + SEM (n=3). P values were obtained using Student's t-test (unpaired 2 tailed, equal variance). The n values indicate the number of independent experiments in all cases.

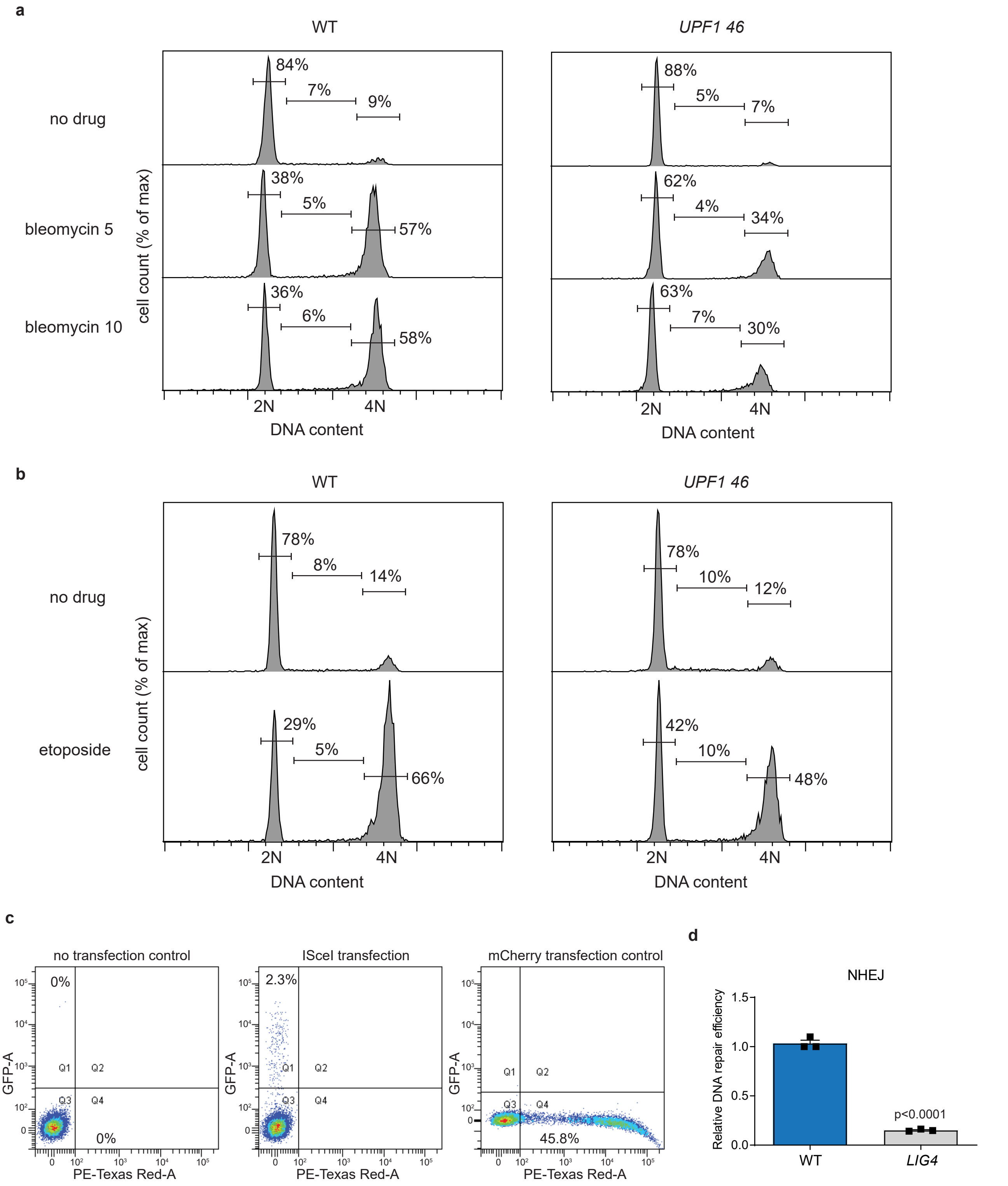

Supplementary Figure 4. UPF1 stimulates repair of non-telomeric DNA damage. a,b Cell cycle analysis of RPE1-hTERT WT and *UPF1* KO cells untreated or treated with 5μg/ml or 10μg/ml bleomycin for 2 days (a) or with 1μM etoposide for 2 days (b) with the level of G1, S and G2/M cells indicated. **c** Flow cytometry data showing induction of GFP expression following transfection of ISceI plasmid into U2OS SA-GFP cells. **d** Analysis of NHEJ activity in HCT116 WT or *LIG4* KO cells. Data plotted are means + SEM (n=3 independent experiments). P values were obtained using Student's t-test (unpaired 2 tailed, equal variance).

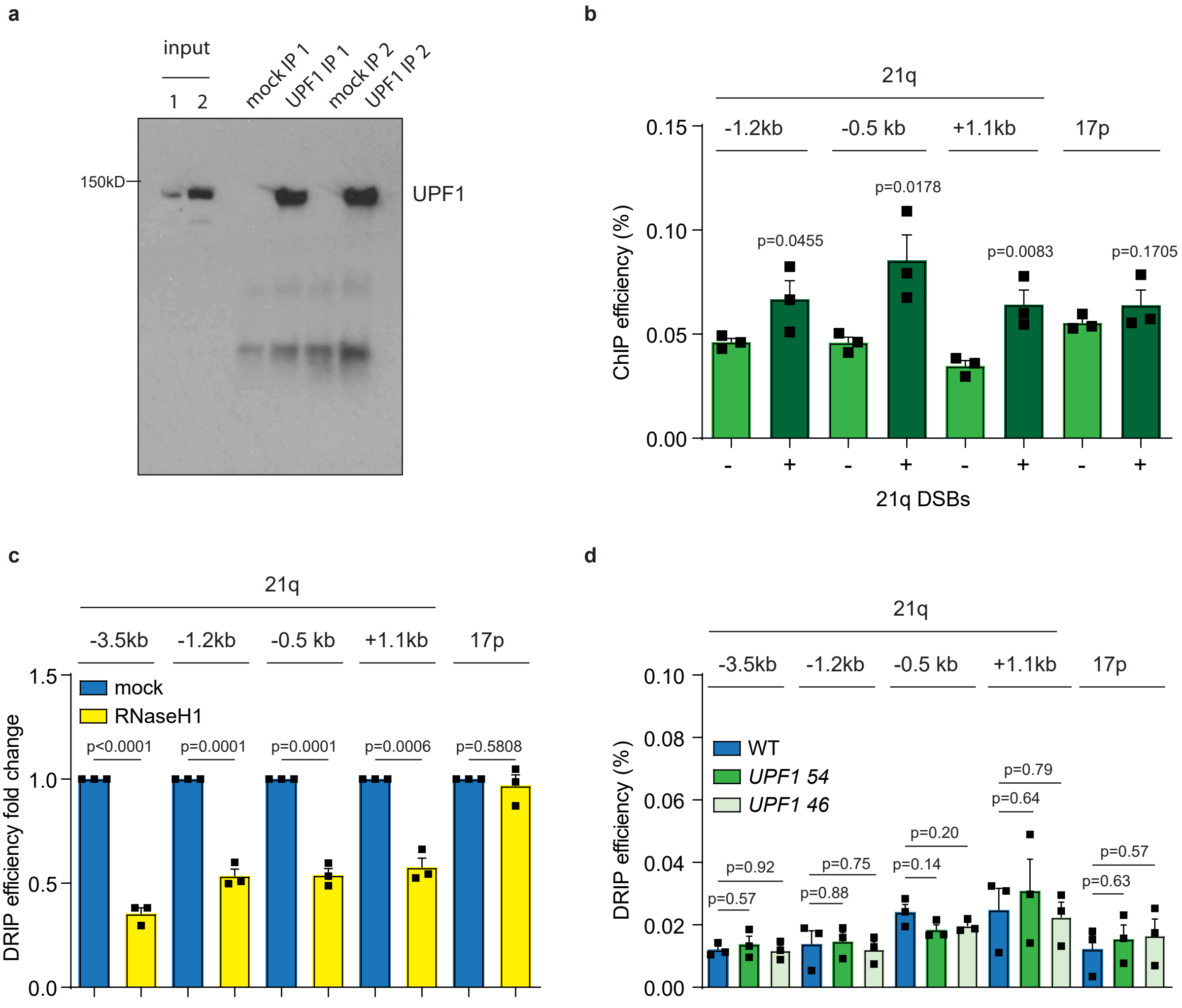

Supplementary Figure 5. UPF1 promotes DNA-RNA hybrid formation at sub-telomeric DSBs. a Immunoprecipitation of UPF1 from RPE1-hTERT cell extracts using anti UPF1 antibody or a mock antibody. Two independent immunoprecipitation experiments are shown. **b** ChIP analysis of UPF1 binding to sub-telomeres with or without DSB induction (at 5 hours after 21q/16p TALEN nucleofection). Data plotted are means + SEM (n=3). P values were obtained using Student's t-test (unpaired 1 tailed, equal variance). **c,d** DRIP analysis showing the levels of DNA-RNA hybrids at the indicted loci in RPE1-hTERT WT cells after in vitro digest with RNaseH1 or mock treated (c) or in RPE1-hTERT WT and *UPF1* KO cells in the absence of DSBs (d). Data plotted are means + SEM (n=3). P values were obtained using Student's t-test (unpaired 2 tailed, equal variance). The n values indicate the number of independent experiments in all cases.

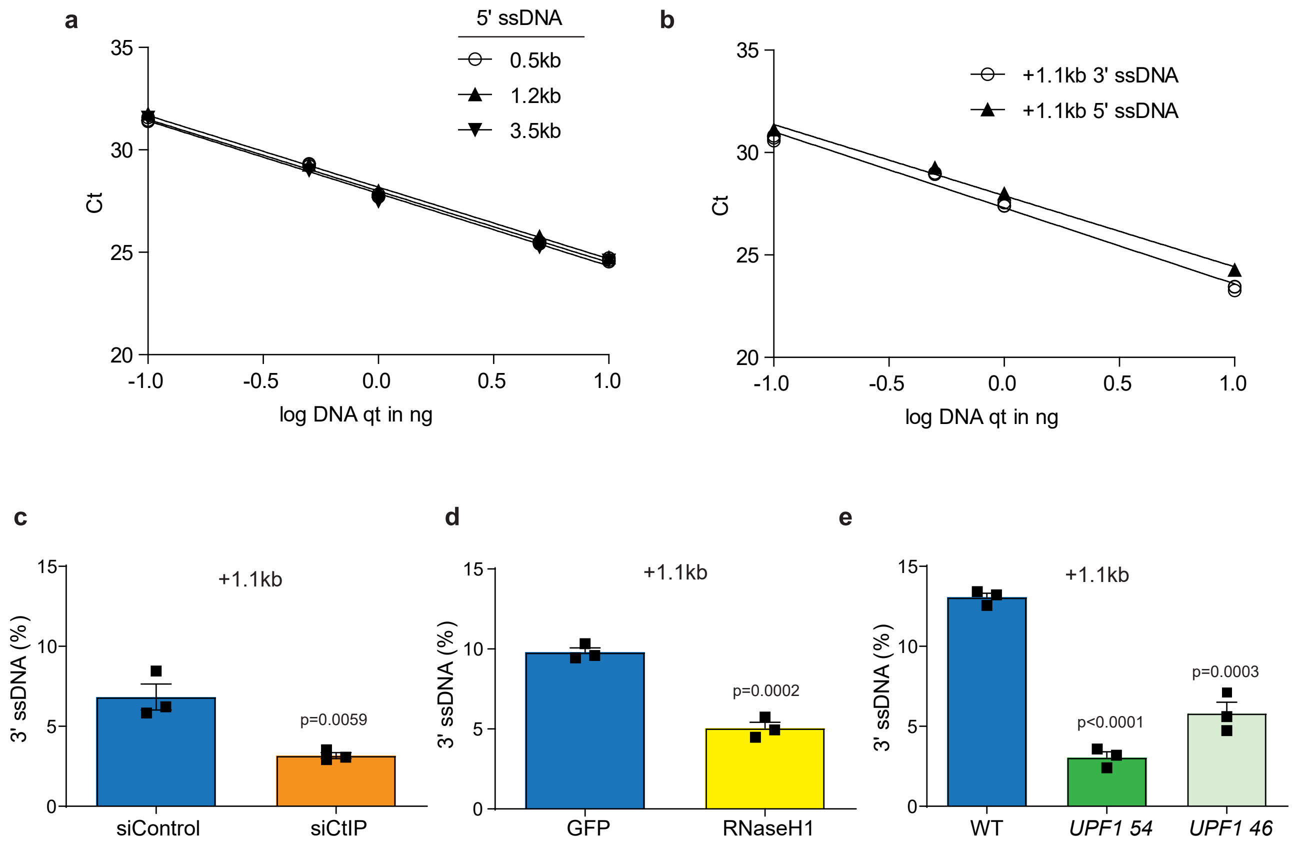

Supplementary Figure 6. UPF1 stimulates R loop formation at sub-telomeric DSBs. a,b Development of QAOS assay in RPE1-hTERT cells. Threshold cycle (Ct) values were obtained from QAOS qPCR for the indicated loci and plotted against the log value of the amount of boiled DNA to generate a standard curve. **c-e** QAOS analysis showing the level of 3′ ssDNA at the indicated loci in RPE1-hTERT cells transfected with CtIP or control siRNAs (c), RPE1-hTERT cells expressing RNaseH1 or GFP (d) and RPE1-hTERT WT and *UPF1* KO cells (e) at 10 hours after 21q/16p TALENs nucleofection. Data plotted are means + SEM (n=3 independent experiments). P values were obtained using Student’s t-test (unpaired 1 tailed, equal variance).

Primer	Sequence (5'-3')
21q1	CTTGGTGTCGAGAGAGGTAG
16p1	TGGACTTCTCACTTCTAGGGCAG
10q21T	GTACTGCCTGCCTTTGGGAT
M449	GTACTGCATGGCTTTGGGAC
Teltail	TGCTCCGTGCATCTGGCATC
Telorette 2	TGCTCCGTGCATCTGGCATCTAACCCT
21q -0.5kb 3' tagging	GATCTCGAGCTCGATATCGGATCCATTGAGGTACATGT
21q -0.5kb 3' forward	AGAGGCAGCTGCAGTAGATACAAACG
21q -0.5kb 3' reverse tag	GATCTCGAGCTCGATATC GGATCCATT
21q -0.5kb 5' tagging	CAGCGCAGCGGCATGAGGCAGCTGCA
21q -0.5kb 5' reverse	TGGGACCTCCTCACTGTTGATTGA
21q -0.5kb 5' forward tag	CAGCGCAGCGGCATGAG
21q -0.5kb 3'/5' Taqman probe	CCGAATCCACTCTGGCACCAGCCTCCTTC
21q -1.2kb 3' tagging	AAGGAGCGCAGCGCCTGTACCACTCCAGCACT
21q -1.2kb 3' forward	CAGGATAGTG GGCTCTGTTAGAGTAGATAGC
21q -1.2kb 3' reverse tag	AAGGAGCGCAGCGCCTGTACCA
21q -1.2kb 5' tagging	CAGCGCAGCGGCATGAGCTCCTGGAAAA
21q -1.2kb 5' reverse	CATCCACTCCAGCACTAGAGATGCTACT
21q -1.2kb 5' forward tag	CAGCGCAGCGGCATGAG
21q -1.2kb 3'/5' Taqman probe	TCTGTGAAGGCTCACCTGGAGGGACCACCA
21q -3.5kb 3' tagging	TGCCCTCGCATCGCTCTCGAAGCATCTGTTTC
21q -3.5kb 3' forward	CCACCCACCCAGTGAGAGATTTATT
21q -3.5kb 3' reverse tag	TGCCCTCGCATCGCTCTCGAA
21q -3.5kb 5' tagging	CAGCGCAG CGGCATGAGATTTATTTTCTA
21q -3.5kb 5' reverse	CCTTTACCTCCTCCTTGAAGCATCTG
21q -3.5kb 5' forward tag	CAGCGCAGCGGCATGAG
21q -3.5kb 3'/5' Taqman probe	TGACAGGCTGGGAAGCATAGCCTCCAGCCA
21q +1.1kb 3' tagging	CAGCGCAGCGGCATGAGTTCTTCTCAG
21q +1.1kb 3' forward	GAATGCTGCTCCGCCTTTACG
21q +1.1kb 3' reverse tag	CAGCGCAGCGGCATGAG
21q +1.1kb 5' tagging	CAGCGCAGCGGCATCTGCTCCGCCTT
21q +1.1kb 5' reverse	GGAGCAGAGTTCTTCTCAGGTCAG
21q +1.1kb 5' forward tag	CAGCGCAGCGGCATCTG
21q +1.1kb 3'/5' Taqman probe	TGCTGAACAGAACACAGCTCCGCCCTCGCA
ch1 dsDNA control forward[1]	TGAGGAGGTGACATTAGAACTCAGA
ch1 dsDNA control reverse[1]	AGGACTCACTTACACGGCCTTT
ch1 dsDNA control Taqman probe[1]	TTGCAAGGCTGCTTCCTTACCATTCAA
17p control forward	CCCGCACCTCCTCACCTCATC
17p control reverse	GCTTAGCTGAAGGGAACATACAGGCTCTT
21p control forward	CTGAAACTGGATCCCTTCCTTAC
21p control reverse	CTTGCCCATGCCTAAGTTCT
UPF1 RTqPCR forward	TGGTTAAGAGACATGCGGCT
UPF1 RTqPCR reverse	CTCATCGCCATAATTATCAGGGACC
UPF2 RTqPCR forward	AGTCAAGCTGAGGGCATCGG
UPF2 RTqPCR reverse	TATGTGACCCAGGACAATCAGCG
UPF3B RTqPCR forward	AAGAAACAAGGATCGTCCAGCG
UPF3B RTqPCR reverse	GCTAATACCACTTTCCTGCTTCC
GADD45B RTqPCR forward	ATCAACATCGTGCGGGTGTC
GADD45B RTqPCR reverse	GTGTGAGGGTTCGTGACCAG
NAT9 RTqPCR forward[2]	GACCCTGGAGCAGGAGTATG
NAT9 RTqPCR reverse[2]	CCACCATGCAGCTCTCTTC
GAPDH RTqPCR forward	CCGCATCTTCTTTTGCGTCG
GAPDH RTqPCR reverse	GCCCAATACGACCAAATCCGT

Supplementary Table 1. Sequences of the primers used in this study.

1 Zhou, Y., Caron, P., Legube, G. & Paull, T. T. Quantitation of DNA double-strand break resection intermediates in human cells. Nucleic Acids Res 42, e19, doi:10.1093/nar/gkt1309 (2014).

2 Tani, H. et al. Identification of hundreds of novel UPF1 target transcripts by direct determination of whole transcriptome stability. RNA Biol 9, 1370-1379, doi:10.4161/rna.22360 (2012).
