# Supplementary material for: UPF1 promotes the formation of R loops to stimulate DNA double-strand break repair
Source: Nat Commun. 2021 Jun 22;12:3849. doi: 10.1038/s41467-021-24201-w (PMC8219777; doi:10.1038/s41467-021-24201-w)
Supplement: Supplementary file 3 — Reporting Summary [file 41467_2021_24201_MOESM3_ESM.pdf]

nature research

Corresponding author(s): Duncan Baird

Last updated by author(s): May 27, 2021

Reporting Summary

Nature Research wishes to improve the reproducibility of the work that we publish. This form provides structure for consistency and transparency in reporting. For further information on Nature Research policies, see our Editorial Policies and the Editorial Policy Checklist.

Statistics

For all statistical analyses, confirm that the following items are present in the figure legend, table legend, main text, or Methods section.

n/a	Confirmed	
☐	☒	The exact sample size (*n*) for each experimental group/condition, given as a discrete number and unit of measurement
☐	☒	A statement on whether measurements were taken from distinct samples or whether the same sample was measured repeatedly
☐	☒	The statistical test(s) used AND whether they are one- or two-sided<br>*Only common tests should be described solely by name; describe more complex techniques in the Methods section.*
☒	☐	A description of all covariates tested
☒	☐	A description of any assumptions or corrections, such as tests of normality and adjustment for multiple comparisons
☐	☒	A full description of the statistical parameters including central tendency (e.g. means) or other basic estimates (e.g. regression coefficient) AND variation (e.g. standard deviation) or associated estimates of uncertainty (e.g. confidence intervals)
☐	☒	For null hypothesis testing, the test statistic (e.g. *F*, *t*, *r*) with confidence intervals, effect sizes, degrees of freedom and *P* value noted<br>*Give P values as exact values whenever suitable.*
☒	☐	For Bayesian analysis, information on the choice of priors and Markov chain Monte Carlo settings
☒	☐	For hierarchical and complex designs, identification of the appropriate level for tests and full reporting of outcomes
☒	☐	Estimates of effect sizes (e.g. Cohen's *d*, Pearson's *r*), indicating how they were calculated

Our web collection on statistics for biologists contains articles on many of the points above.

Software and code

Policy information about availability of computer code

Data collection	NC-3000™ NucleoView v2.1 (Chemometec)<br>BD FACSDiva™ 8.0.1 Software (BD Biosciences)<br>ImageQuant TL Software v8.1 (GE Healthcare)<br>ViiA™ 7 v1.2.3 Software (Thermo Fisher Scientific)<br>QuantStudio v1.3 Software (Thermo Fisher Scientific)
Data analysis	NC-3000™ NucleoView v2.1 (Chemometec)<br>GraphPad Prism 5 (GraphPad Software)<br>BD FACSDiva™ 8.0.1 Software (BD Biosciences)<br>Flowjo v10 (BD Biosciences)<br>Applied Biosystems qPCR Analysis Modules (Thermo Fisher Scientific)<br>Image J v1.52, open source:https://imagej.nih.gov/ij/index.html<br>ImageQuant TL Software v8.1 (GE Healthcare)<br>Microsoft Excel v16 (Microsoft)<br>Adobe Photoshop CS v5.1 (Adobe)<br>CRISPR Finder online tool (Wellcome Sanger Institute Genome Editing website)

For manuscripts utilizing custom algorithms or software that are central to the research but not yet described in published literature, software must be made available to editors and reviewers. We strongly encourage code deposition in a community repository (e.g. GitHub). See the Nature Research guidelines for submitting code & software for further information.

Data

Policy information about availability of data

All manuscripts must include a data availability statement. This statement should provide the following information, where applicable:

- Accession codes, unique identifiers, or web links for publicly available datasets
- A list of figures that have associated raw data
- A description of any restrictions on data availability

All data generated in this study are included in this article. Source data are provided with this paper.

Field-specific reporting

Please select the one below that is the best fit for your research. If you are not sure, read the appropriate sections before making your selection.

☒ Life sciences ☐ Behavioural & social sciences ☐ Ecological, evolutionary & environmental sciences

For a reference copy of the document with all sections, see nature.com/documents/nr-reporting-summary-flat.pdf

Life sciences study design

All studies must disclose on these points even when the disclosure is negative.

Sample size	Sample-size calculation were not performed. Experiments were repeated for 2-5 times, based on similar studies in the field below:<br><br>D'Alessandro, G. et al. BRCA2 controls DNA:RNA hybrid level at DSBs by mediating RNase H2 recruitment. Nat Commun 9, 5376, doi:10.1038/s41467-018-07799-2 (2018).<br>Cohen, S. et al. Senataxin resolves RNA:DNA hybrids forming at DNA double-strand breaks to prevent translocations. Nat Commun 9, 533, doi:10.1038/s41467-018-02894-w (2018).
Data exclusions	qPCR value for one of the triplicate reaction from a single sample were treated as outlier and excluded if it differs by >1 Ct from other duplicates or if it differs by >0.5 Ct from other duplicates with superimposable amplification curves. This exclusion criteria were pre-establised before data analysis and serves to reduce experimental error introduced by pipetting error.
Replication	The experiments were repeated for 2-5 times as indicated independently to confirm reproducibility. All experiments were replicated successfully except fig. 3F (siUPF3B cells grew slower than expected and there were not enough cells for TALEN transfection), fig. 4D (UPF1 54 cells grew slower than expected and there were not enough cells for the experiment), and fig.6B (DNA yield was very low for the 5 hour time point sample and there were not enough DNA for setting up qPCR reaction).
Randomization	Cells were randomly allocated for the modulation of gene activity or the induction of DNA damage.
Blinding	Blinding was not performed during data collection or analysis. This is because all data were collected by machines and analysis were performed by using programs and protocols which treat all samples equally, so it is unlikely that bias can be introduced.

Reporting for specific materials, systems and methods

We require information from authors about some types of materials, experimental systems and methods used in many studies. Here, indicate whether each material, system or method listed is relevant to your study. If you are not sure if a list item applies to your research, read the appropriate section before selecting a response.

Materials & experimental systems

n/a	Involved in the study	
☐	☒	Antibodies
☐	☒	Eukaryotic cell lines
☒	☐	Palaeontology and archaeology
☒	☐	Animals and other organisms
☒	☐	Human research participants
☒	☐	Clinical data
☒	☐	Dual use research of concern

Methods

n/a	Involved in the study	
☒	☐	ChIP-seq
☐	☒	Flow cytometry
☒	☐	MRI-based neuroimaging

Antibodies

Antibodies used	anti-phospho-RPA32 (S4/S8) rabbit antibody, A300-245A-M, Cambridge Biosciences, clone name: polyclonal, Lot number:#8<br>anti-phospho-Chk1 (Ser345) rabbit antibody, 2348, Cell signalling, clone name: 133D3, Lot number:18

anti phospho-Chk2 (Thr68) rabbit antibody, 2661, Cell signalling, clone name: polyclonal, Lot number:11
anti-UPF1 goat antibody, A300-038A, Cambridge Biosciences, clone name: polyclonal, Lot number:#4
anti-UPF1 mouse antibody, sc-393594, Santa Cruz, clone name: C-6, Lot number:#L0513
anti-UPF3B rabbit antibody, A303-688A, Cambridge Biosciences, clone name: polyclonal, Lot number: #1
anti-actin rabbit antibody, A2066, Sigma-Aldrich, clone name: polyclonal, Lot number:#058M4812V
anti-ATM(S1981) mouse antibody , 200-301-400S, Cambridge Bioscience, clone name: 10H11.E12, Lot number:32182
anti-CHK1 mouse antibody, 2360S, Cell Signalling, clone name: 2G1D5, Lot number:3
anti-CHK2 rabbit antibody, ab109413, Abcam, clone name: EPR4325, Lot number:GR45022-15
anti-RPA32 rabbit antibody, A300-244A-M, Cambridge Bioscience, clone name:polyclonal, Lot number:#3
anti-ATM goat antibody, A300-136A-T, Cambridge Bioscience, clone name: polyclonal, Lot number:#2

Validation

anti-phospho-RPA32 (S4/S8) rabbit polyclonal antibody (A300-245A-M, Cambridge Biosciences): validated by supplier and publications, see websites
https://www.bioscience.co.uk/product~696934
https://www.bethyl.com/product/A300-245A/Phospho+RPA32+(S4+S8)+Antibody

anti-phospho-Chk1 (Ser345) rabbit monoclonal antibody (2348, Cell signalling): validated by supplier and publications, see websites
https://www.cellsignal.co.uk/products/primary-antibodies/phospho-chk1-ser345-133d3-rabbit-mab/2348
https://www.citeab.com/antibodies/123248-2348-phospho-chk1-ser345-133d3-rabbit-mab

anti phospho-Chk2 (Thr68) rabbit polyclonal antibody (2661, Cell signalling): validated by supplier and publications, see websites
https://www.cellsignal.co.uk/products/primary-antibodies/phospho-chk2-thr68-antibody/2661
https://www.citeab.com/antibodies/123816-2661-phospho-chk2-thr68-antibody

anti-UPF1 goat polyclonal antibody (A300-038A, Cambridge Biosciences): validated by supplier and publications, see websites
https://www.bioscience.co.uk/product~69341
https://www.bethyl.com/product/A300-038A/RENT1+Antibody

anti-UPF1 mouse monoclonal antibody (sc-393594, Santa Cruz): validated by supplier and publications, see website
https://www.scbt.com/p/rent1-antibody-c-6

anti-UPF3B rabbit polyclonal antibody (A303-688A, Cambridge Biosciences): validated by supplier and publications, see websites
https://www.bioscience.co.uk/product~523850
https://www.bethyl.com/product/A303-688A/UPF3B+Antibody

anti-actin rabbit polyclonal antibody (A2066, Sigma-Aldrich): validated by supplier and publications, see website
https://www.sigmaaldrich.com/catalog/product/sigma/a2066?lang=en&region=GB

anti-ATM(S1981) mouse antibody , (200-301-400S, Cambridge Bioscience): validated by supplier and publications, see website
https://rockland-inc.com/Product.aspx?id=41061

anti-ATM goat antibody, (A300-136A-T, Cambridge Bioscience): validated by supplier and publications, see website
https://www.bethyl.com/product/A300-136A/ATM+Antibody

anti-CHK1 mouse antibody, (2360S, Cell Signalling), validated by supplier and publications, see website
https://www.cellsignal.co.uk/products/primary-antibodies/chk1-2g1d5-mouse-mab/2360

anti-CHK2 rabbit antibody, (ab109413, Abcam) validated by supplier and publications, see website
https://www.abcam.com/Chk2-antibody-EPR4325-ab109413.html

anti-RPA32 rabbit antibody, (A300-244A-M, Cambridge Bioscience) validated by supplier and publications, see website
https://www.bethyl.com/product/A300-244A/RPA32+Antibody

Eukaryotic cell lines

Policy information about cell lines

Cell line source(s)

RPE1-hTERT human retinal pigment epithelial cell line from Prof. Eric Hendrickson (University of Minnesota, USA), originally from ATCC (Manassas)
HCT116 human colorectal carcinoma cell line from Prof. Eric Hendrickson (University of Minnesota, USA), originally from ATCC (Manassas)
U2OS human bone osteosarcoma cell line from Prof. Jeremy Stark (City of Hope Comprehensive Cancer Center, USA), originally from ATCC (Manassas)

Authentication

RPE1-hTERT human retinal pigment epithelial cell line were authenticated by showing similar morphology to RPE1-hTERT cells as shown on ATCC website.

HCT116 human colorectal carcinoma cell lines were authenticated in the lab by showing similar morphology to HCT116 cells as shown on ATCC website and by having expected DNA repair deficiency as described in the publication below:
https://genome.cshlp.org/content/early/2016/03/03/gr.200840.115

U2OS human bone osteosarcoma reporter cell lines were authenticated by showing similar morphology to U2OS cells as shown on ATCC website and by the production of GFP positive cells following the transfection of plasmids expressing ISceI as described in the publication below:

	https://link.springer.com/protocol/10.1007%2F978-1-61779-998-3_27
Mycoplasma contamination	PCR test were performed when these cell lines were received and showed that all cell lines were mycoplasma negative. Test were repeated regularly which showed that all cell lines remained mycoplasma negative during the course of this study.
Commonly misidentified lines (See ICLAC register)	none

Flow Cytometry

Plots

Confirm that:

☒ The axis labels state the marker and fluorochrome used (e.g. CD4-FITC).

☒ The axis scales are clearly visible. Include numbers along axes only for bottom left plot of group (a 'group' is an analysis of identical markers).

☒ All plots are contour plots with outliers or pseudocolor plots.

☒ A numerical value for number of cells or percentage (with statistics) is provided.

Methodology

Sample preparation	U2OS human bone osteosarcoma reporter cells (untrasfected or transfected with ISceI or mCherry expressing plasmids) were tripsinised, washed once in PBS, resuspended in PBS and subjected to flow cytometry.
Instrument	BD LSRFortessa™ cell analyser (BD Biosciences)
Software	BD FACSDiva™ 8.0.1 Software (BD Biosciences)
Cell population abundance	Not applicable- experiments were performed on a single cell line.
Gating strategy	Forward versus side scatter (FSC vs SSC) and forward scatter height versus forward scatter area (FSC-H vs FSC-A) plots were used to exclude cell debris and doublets. Gating used to detect cells expressing GFP or mCherry are shown in Supplementary Fig. 4c

☒ Tick this box to confirm that a figure exemplifying the gating strategy is provided in the Supplementary Information.
